# Community treatment of latent tuberculosis in child and adult refugee populations: outcomes and successes

**DOI:** 10.3389/fpubh.2023.1225217

**Published:** 2023-10-23

**Authors:** Emily Harwood-Johnson, Karen S. Leis, Jacelyn Hanson, Jordan Olfert, Yvonne Blonde, Mahli Brindamour

**Affiliations:** ^1^College of Medicine, University of Saskatchewan, Saskatoon, SK, Canada; ^2^Department of Pediatrics, College of Medicine, University of Saskatchewan, Saskatoon, SK, Canada; ^3^Department of Family Medicine, College of Medicine, University of Saskatchewan, Saskatoon, SK, Canada; ^4^Department of Respirology, College of Medicine, University of Saskatchewan, Saskatoon, SK, Canada; ^5^Department of Academic Family Medicine, College of Medicine, University of Saskatchewan, Saskatoon, SK, Canada

**Keywords:** latent tuberculosis, immigrant, refugee, Canada, community

## Abstract

**Background:**

Tuberculosis (TB) is the world’s leading infectious cause of death, killing millions every year. In Canada, considered a low-incidence country for TB, the burden of the disease is unequally distributed, with most cases of latent tuberculosis infection (LTBI) experienced by newcomers from endemic regions. The purpose of this study was to measure LTBI treatment acceptance and completion outcomes of LTBI treatment at the REACH clinic in Saskatoon, a local refugee clinic providing primary care-based LTBI management.

**Methods:**

A retrospective case series by sampling methodology was applied to review patients who visited the REACH clinic between January 2017 and June 2021 and who had an interferon-gamma release assay (IGRA) or tuberculin skin test (TST) done for LTBI screening. Those with positive results were retained for analysis. The LTBI treatment acceptance and completion groups were compared according to demographic variables, WHO regions of origin, year of arrival to Canada, and LTBI treatment regimen.

**Results:**

A total of 523 patients were screened for LTBI, of whom 125 tested positive, leading to a test positivity of 23.9%. The treatment acceptance rate was 84.8%, and the treatment completion rate was 93.3%. All of those who declined treatment were more than 18 years of age (*p* = 0.02). Otherwise, treatment acceptance and completion rates did not vary significantly in association with gender, categories of refugees, WHO region of origin, year of arrival to Canada, or LTBI treatment regimen used.

**Discussion:**

The refugee clinic acceptance and completion rates in this study are high and meet Canadian TB standards of care. The multidisciplinary clinic model and community support are important facilitators, which, in combination with shorter treatment regimens, offer a path forward for LTBI management among refugees resettling in low-incidence countries.

## 1. Introduction

Once thought a “romantic disease” (1) and long since forgotten in popular culture, tuberculosis (TB) continues to be the world’s leading infectious cause of death that killed 1.4 million people in 2021 (2). With its latest general population incidence rate being 5.3 active TB cases per 100,000 in 2021, Canada is considered a low-incidence country and has been since the 1980s (3, 4). While incidence numbers are indeed relatively low within the general Canadian population, they do not reflect the deep inequities in the distribution of the TB burden (5). The vast majority of new TB infection diagnoses each year are among newcomers to Canada and Indigenous people, outlining how tuberculosis remains a disease of poverty, targeting marginalized populations disproportionally (5–7).

In 2001, Canada adopted the *Stop TB Partnership* goal of eliminating TB as a public health threat by 2030 (8), and in 2014, the country signed the World Health Organization (WHO)‘s *Action Framework for Low-Incidence Countries* for the pre-elimination goal of less than 1 TB case per 100,000 by 2035 (2, 9). Part of the path outlined by the WHO to reduce tuberculosis impact and eventually reach TB elimination in low-incidence countries such as Canada includes the detection and management of latent tuberculosis infection (LTBI) among higher-risk groups (10–13). Indeed, in low-incidence countries, most new active TB cases are due to the reactivation of LTBI. This makes people living with LTBI, while non-contagious, a reservoir for future active TB cases and possible subsequent transmission (13). For reference, a summary of the major differences between LTBI and active TB is outlined in Table 1.

**Table 1:** Major differences between LTBI and active TB.

TB spectrum of disease	LTBI	Active TB
Clinical presentation	Asymptomatic	Symptomatic
Imaging	N or granuloma on CXR	Abnormal
Screening tests (IGRA or TST)	+	Usually + can be −
Smear results	−	+
Culture results	−	+
Person-to-person transmission	No	Yes*
Possible management approaches	1HP, 3HP, 3HR, 4R, 6H, 9H	2HRZ(E)/4HR, others

* Only with pulmonary or laryngeal involvement.

TB, tuberculosis; LTBI, latent tuberculosis infection; N, normal; CXR, chest X-ray; IGRA, interferon gamma release assay; TST, tuberculin skin test; 1HP, 1 month daily isoniazid-rifapentine; 3HP, once weekly isoniazid-rifapentine for 12 weeks; 4R, 4 months daily rifampin; 6H, 6 months daily isoniazid; 9H, 9 months daily isoniazid; 2HRZ(E)/4HR, 2 months isoniazid + rifampin + pyrazinamide ±ethambutol followed by 4 months of isoniazid + rifampin.

Between 2013 and 2020, 70–72% of TB cases diagnosed in Canada were among individuals born outside of the country, and 42% of those cases occurred within 5 years of arrival (4, 5). For a variety of reasons, including a higher likelihood of being born in an area of high incidence and migrating through precarious conditions, refugees are at increased risk of tuberculosis infection compared to other newcomer categories (such as certain skilled workers or international students) (14). The most recent 2022 Canadian tuberculosis guidelines recommend targeted LTBI screening for refugees aged up to 65 years (15). In the absence of a formal national immigration LTBI screening and management program in Canada, the management of LTBI among refugees varies by province and is left to the discretion of healthcare providers (14, 16).

In Saskatchewan (SK), TB management (including LTBI) has been centralized through the provincial Tuberculosis Prevention and Control (TBPC) program since the 1980s (17). Until the establishment of the Refugee Engagement and Community Health (REACH) clinic in 2017, all TB regimen prescriptions were done exclusively by TBPC’s TB consultants, and no specific TB program for refugees existed. The REACH clinic is housed within the Saskatoon Community Clinic, which, since its inception in 1962, has been instrumental in the development of Medicare in Canada and in providing access to care to marginalized populations (18). REACH’s mandate is to provide all refugees resettling in Saskatoon with primary and pediatric healthcare through multidisciplinary teams (including interpreters, settlement agencies, and other community partners) for a duration of 12–18 months post-arrival to Canada. The clinic is staffed by providers with additional training in refugee health, trauma-informed care, and infectious diseases. With a population of 266,141 in 2021 (19), Saskatoon is Saskatchewan’s largest city and receives an average of 250–500 refugees per year, depending on world events and the Canadian federal government’s commitment to refugee resettlement.

The REACH clinic is thus well positioned to perform LTBI screening and management for refugees in Saskatoon. Since its creation in 2017, it has worked collaboratively with the province’s TBPC program to design an LTBI program dedicated to this population. REACH’s physicians screen patients as per the Canadian TB standards (15) and manage identified LTBI cases within the clinic, allowing for community-based LTBI treatment and follow-up. Later, REACH patients are followed for LTBI at their primary care home and by their own physician, with TB medications dispensed by the REACH clinic’s partner pharmacy located in the same building. In accordance with national and provincial guidelines (20), TB treatment and support are provided free of charge regardless of the patients’ insured status or immigration documentation (21).

In this study, the primary purpose was to assess the outcomes of the REACH clinic’s LTBI community treatment program in terms of treatment acceptance and completion.

## 2. Context

### 2.1. Setting and population

The REACH clinic’s patient load from January 2017 to June 2021 was used as a framework and included government-assisted refugees (GARs), private-sponsored refugees (PSRs), and refugee claimants, representing not only the clinic’s sole served population but also the three categories of refugee resettlement in Canada (22). REACH’s patients are screened for LTBI if they were born or have lived in regions of high TB incidence (cut-off of 50 smear-positive pulmonary TB cases per 100,000) (6). Because no TB screening tests are validated in patients under 6 months of age, infants aged less than 6 months were not included in the study (23). During their first clinic visit, all such eligible patients under 2 years of age received a tuberculin skin test (TST), and those over 2 years of age underwent an interferon-gamma release assay (IGRA). Table 2 provides further details regarding the TB screening tests used, the rationale for their choice, and their respective interpretation.

**Table 2:** Screening tests for latent tuberculosis infection used in this study.

Screening test used	IGRA	TST
Screening test method	QuantiFERON-TB Gold Plus 4-tube assay	Mantoux method
Rationale for test choice*	Patients aged ≥ 2 years	Patients aged ≥ 6 months to < 2 years
Interpretation	Positive, negative, or indeterminate** The qualitative result is based on the interpretation of four values (NIL, MITOGEN minus NIL, TB1 minus NIL, and TB2 minus NIL) as per provincial laboratory standards	Negative if < 5 mm Positive if ≥ 10 mm Positive if 5–9 mm in certain higher-risk situations***

* As per the 2022 Canadian Tuberculosis Guidelines (24), while both IGRAs and TSTs are acceptable alternatives as LTBI screening methods, IGRAs can be used for individuals above 2 years of age (23). At the REACH clinic, IGRAs are used preferentially over TSTs for practical reasons (no need for a second visit and good availability of the test, which can be performed on-site and included with other screening tests done at every patient’s initial clinic visit).

** If the IGRA is indeterminate, TST is obtained.

*** High-risk situations include people living with HIV, known recent contact with a patient with known infectious TB, fibronodular disease on CXR, stage 4 and 5 chronic kidney disease prior to organ transplantation, and the receipt of biologics or other immunosuppressive drugs.

### 2.2. Rationale for LTBI treatment regimens used at REACH

There are several treatment regimens for LTBI in Canada, but in recent years, shorter regimens such as 3HP (12 weeks of weekly isoniazid and rifapentine) and 4R (4 months of daily rifampin) are being favored over the lengthier 9H (9 months of daily isoniazid). The 3HR regimen (3 months of daily isoniazid and rifampin) is an acceptable alternative if the previously mentioned regimens are not feasible (20). Despite its 2014 approval by the United States Food and Drug Administration (FDA) for LTBI treatment in 2014, rifapentine was difficult to obtain in Canada until 2018 and remains only available through the Urgent Public Health Need regulation (25). The use of 3HP in Canada is bound by the obligation to use directly observed therapy (DOT), which is not the case for other available LTBI treatments. The REACH clinic’s physicians received approval from the TBPC to prescribe and use 3HP with DOT in January 2020. Prior to this, the TBPC program oversaw all of these cases centrally, and several of REACH’s patients were referred out of primary care to be able to use this shorter regimen. Nonetheless, from 2017 to 2020, most of REACH’s LTBI cases were managed with 4R due to administrative burdens and associated delays in obtaining rifapentine. However, a transition was made to 3HP and DOT for most cases without contraindications in early 2020. This said, a significant decrease in patient numbers during the COVID-19 pandemic (following the reduction in international travel and subsequent decrease in refugees entering the country) led to very few patients being managed with 3HP during the study period. In addition, a shortage of rifampin in Canada from July 2019 to early 2021 led to the increased use of the 9H or 3HR regimens at the REACH clinic due to the difficulty in accessing either or both rifampin and rifapentine during that time period (26). Patients with LTBI under 2 years of age were treated with 4R when rifampin was available, and no contraindications were present, or 9H in other instances.

## 3. Materials and methods

Ethical approval for this project was received from the University of Saskatchewan Biomedical Ethics Board (Bio-REB ID number 2616).

### 3.1. Data collection

An electronic coding manual, including a data collection tool, was created. Cases were identified for sampling following the convenience method (27). All of the REACH clinic charts that were opened between 1 January 2017 and 30 June 2021 were identified and pulled from MedAccess, the electronic medical record (EMR) used at the clinic. The supervising author (MB), who, as part of the REACH clinic’s clinical team, had access to the EMR system, manually identified all charts from this period containing testing for IGRAs and TSTs and verified that no charts were missing by automatically pulling MedAccess charts containing the keywords IGRA, QuantiFERON Gold, TST, and Mantoux. These charts were then searched further by the first author (EHJ) to identify those containing positive IGRA and TST results. EHJ, who holds an M.Sc. in Community Health and Epidemiology, was specifically trained for data collection by the co-authors (MB, KL, JH). To form the master list, EHJ reviewed charts with positive LTBI screening test results to further identify patients who were offered LTBI treatment within the REACH clinic LTBI program. EHJ used the electronic coding manual to obtain demographic and clinical data from charts included in the master list, and those were subsequently coded for de-identification. Table 3 outlines the demographic characteristics of the study population. Clinical data included in the data collection tool and used for this study included LTBI screening test types and results, LTBI treatment regimen chosen for each patient, timeframe to treatment completion, completion outcome, and reason(s) for non-completion, if applicable.

**Table 3:** LTBI treatment acceptance and outcome by LTBI treatment used and demographic characteristics.

Demographic characteristics *n* (%)		Treatment acceptance			Treatment outcome		
		Yes *n* (%)	No *n* (%)	*p*-value	Completed *n* (%)	Not completed *n* (%)	*p*-value
Age < 18 years > 18 years Total	21 (31.8) 45 (68.2) 66 (100)	21 (100) 35 (77.8) 56 (84.9)	0 (0) 10 (22.2) 10 (15.1)	0.02^b^	17 (94.4) 25 (95.6) 42 (93.33)	1 (5.6) 2 (7.4) 3 (6.7)	1.000^b^
Gender Female Male Non-specified/other Total	30 (45.5) 36 (54.5) 0 (0) 66 (100)	25 (83.3) 31 (86.1) 0 (0) 56 (84.9)	5 (16.7) 5 (13.9) 0 (0) 10 (15.1)	1.00^a^	20 (95.2) 22 (91.7) 0 (0) 42 (93.33)	1 (4.8) 2 (8.3) 0 (0) 3 (6.7)	1.000^a^
WHO Regions* Africa Eastern Mediterranean Southeast Asia South America Total	37 (56.1) 26 (39.4) 2 (3) 1 (1.5) 66 (100)	30 (81.1) 23 (88.5) 2 (100) 1 (100) 56 (84.9)	7 (18.9) 3 (11.5) 0 (0) 0 (0) 10 (15.1)	0.69^a^	23 (92) 16 (94.2) 2 (100) 1 (100) 42 (93.3)	2 (8) 1 (5.9) 0 (0) 0 (0) 3 (6.7)	1.000^a^
Refugee category GARs PSRs Refugee claimants Total	52 (78.8) 10 (15.2) 4 (6) 66 (100)	43 (82.7) 10 (100) 3 (75) 56 (84.9)	9 (17.3) 0 (0) 1 (25) 10 (15.1)	0.25^a^	33 (91.7) 6 (100) 3 (100) 42 (93.3)	3 (8.3) 0 (0) 0 (0) 3 (6.7)	1.000^a^
Arrival year 2017 2018 2019 2020** 2021 Total	30 (45.4) 17 (25.7) 9 (13.7) 9 (13.7) 1 (1.5) 66 (100)	24 (80) 13 (76.5) 9 (100) 9 (100) 1 (100) 56 (84.9)	6 (20) 4 (23.5) 0 (0) 0 (0) 0 (0) 10 (15.1)	0.34^a^	20 (90.9) 13 (100) 6 (85.7) 3 (100) 0 (0) 42 (93.3)	2 (9.1) 0 (0) 1 (14.3) 0 (0) 0 (0) 3 (6.7)	0.52^a^
LTBI treatment chosen n (%)					Completed *n* (%)	Not completed *n* (%)	*p*-value
3HP 3HR 9H 4R Total	2 (4.4) 3 (6.7) 28 (62.2) 12 (26.7) 45 (100)				2 (100) 3 (100) 26 (92.9) 11 (91.7) 42 (93.3)	0 (0) 0 (0) 2 (7.1) 1 (8.3) 3 (6.7)	0.47^a^

* Countries of origin regrouped by WHO regions for clarity but included Eritrea, Somalia, Sudan, Burundi, Syria, Ethiopia, the Democratic Republic of Congo, Kenya, Columbia, Uganda, Tanzania, Nepal, Syria, Niger, Thailand, Pakistan, and Iraq.

** Significant decrease in the number of new arrivals after March 2020 due to the COVID-19 pandemic. Numbers increased back to pre-pandemic levels in the last quarter of 2021 and early 2022 after the study period.

a Fisher–Freema–Halton Test.

b Fisher’s Exact Test.

WHO = World Health Organization, GARs = Government-Assisted Refugees, PSRs = Private-Sponsored Refugees.

### 3.2. Participants

**Figure 1:**
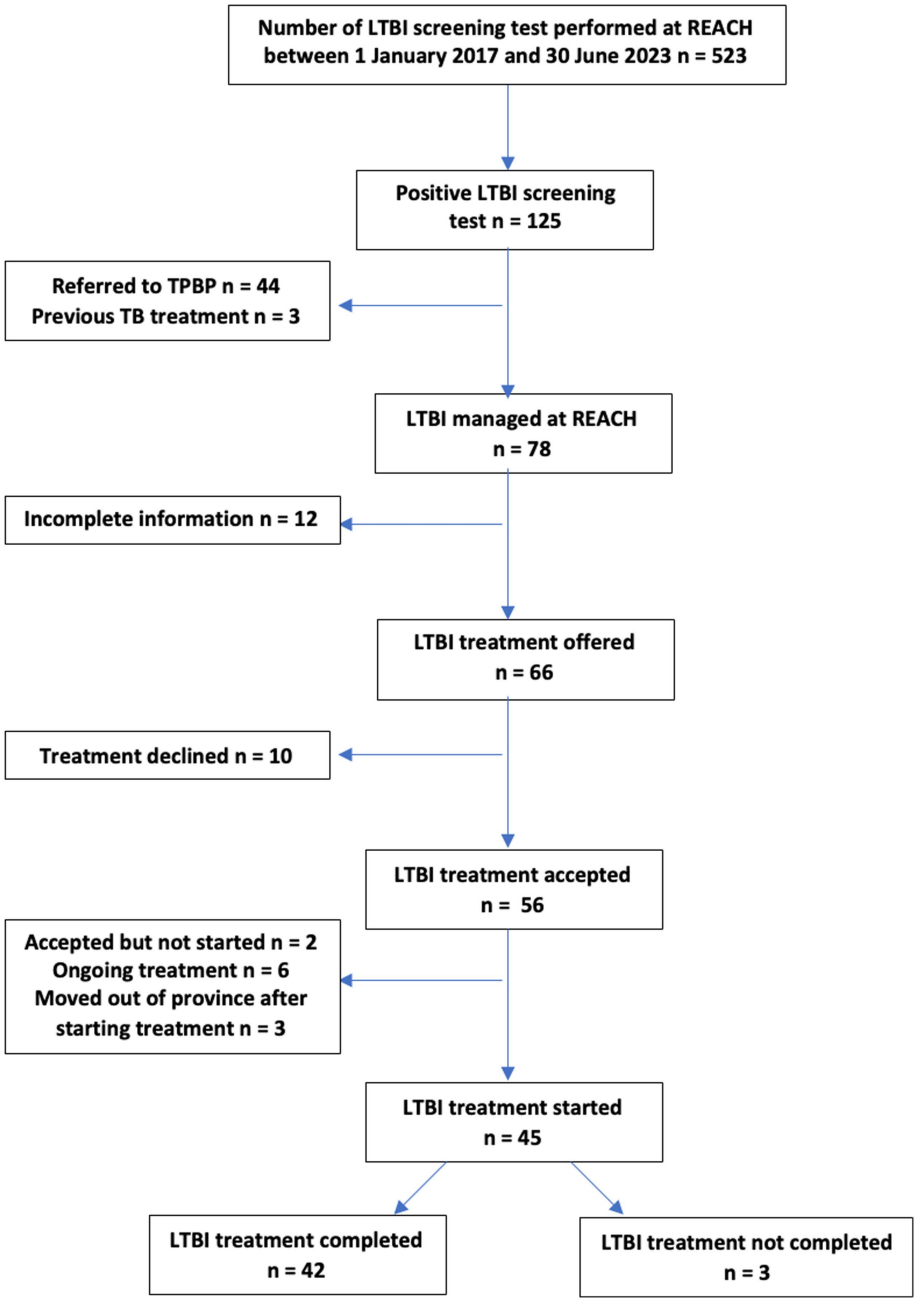
Cascade of care for LTBI management at REACH.

Our study population consisted of patients who were seen at the REACH clinic between 1 January 2017 and 30 June 2021 who had a TB screening test done with a subsequent positive result and who were offered LTBI treatment. During that time, 523 patients had undergone LTBI screening tests, and 125 were positive. Of the 125 REACH patients with a positive LTBI screening test result, 3 were not offered treatment as they had previously completed LTBI prophylaxis or active TB treatment prior to resettlement. Of the remaining 122 patients, 44 were seen prior to the 2020 introduction of 3HP DOT within the REACH clinic and preferred this specific regimen, so they were referred to TBPC for further management without REACH’s involvement in their LTBI care. A total number of 78 patients were included in the final chart review. Figure 1 outlines the REACH clinic’s LTBI cascade of care leading to examined outcomes (treatment acceptance and completion). Of note, none of the patients with a positive LTBI screening test during the study period were found to have active TB.

### 3.3. LTBI treatment completion definitions

LTBI treatment was considered complete if the patient successfully took all of their respective treatment’s prescribed doses within a specific timeframe, which varied according to the selected regimen. Table 4 outlines the LTBI treatment regimens used at REACH during the study period and their respective treatment completion criteria.

**Table 4:** LTBI treatment regimen used at REACH and associated definitions of treatment completion.

LTBI treatment regimen	Agent(s)	Dosage frequency	DOT vs SAT	Duration	Number of doses required for treatment completion with an acceptable timeframe during which treatment completion was considered obtained (28)
3HP	Isoniazid + rifapentine	weekly	DOT	12 weeks	12 (at least 11 doses within 16 consecutive weeks)
4R	rifampin	daily	SAT	4 months	120 (within 6 consecutive months)
9H	isoniazid	daily	SAT	9 months	270 (within 12 consecutive months)
3HR	Isoniazid + rifampin	daily	SAT	3 months	90 (within 4 consecutive months)

H, isoniazid; P, rifapentine; R, rifampin; DOT, directly-observed therapy; SAT, self-administered therapy.

LTBI treatment outcomes (treatment acceptance and subsequent treatment completion) were compared by demographic variables and the LTBI treatment regimen chosen, where applicable.

### 3.4. Statistical analysis

A retrospective case series by sampling methodology was applied for data analysis, where only exposed patients were included. As such, a statistical analysis of risk was not possible (29). Patients with a positive LTBI screening test result who were offered treatment formed the exposure group. The outcomes examined were LTBI treatment acceptance (accepted vs. declined) and completion (completed vs. not completed). The Fisher exact test and Fisher–Freeman–Halton were applied to determine statistically significant differences between both the treatment acceptance groups and the treatment completion groups.

As a secondary goal, the screening test positivity for IGRAs and TSTs was calculated. The total number of positive tests was used as a numerator, and the total number of patients for whom an LTBI screening test was performed was used as a denominator. Demographic and detailed clinical data were not collected for this larger group as this exceeded the scope of our study.

The Office of the Vice Dean of Research of the College of Medicine of the University of Saskatchewan allowed for statistician support and review of the statistical analysis.

## 4. Results

Of the 78 patients who were tested for LTBI, those who had a positive test result, those who did not receive TB treatment prior to resettlement, and those who were not transferred to TBPC, *n* = 12 (15.3%) were not included in the final chart review due to missing information on treatment acceptance and outcome. Missing information was due to moving out of the province prior to discussing management options, deferring treatment due to breastfeeding or pill burden due to other conditions and not knowing if treatment was accepted later on, awaiting further investigation prior to offering LTBI treatment, or unknown reasons.

Of the remaining 66 patients offered LTBI treatment, *n* = 56 (84.9%) accepted LTBI treatment, and *n* = 10 (15.1%) declined treatment to opt for medical surveillance. Reasons for declining treatment included concerns regarding side effects related to pregnancy or non-specified. All patients who declined treatment were adults (age > 18 years old), leading to a statistically significant difference (*p* = 0.02) between adults and children (age < 18 years old) when it came to treatment acceptance. Treatment acceptance did not vary significantly in association with gender, categories of refugees, WHO region of origin, or year of arrival to Canada (*p* >0.05).

Of those 56 patients who chose and initiated treatment, *n* = 3 (5.3%) moved out of the province before treatment completion and were transferred to other provincial TB programs, *n* = 2 (3.5%) had accepted the treatment but not started prior to study completion, and *n* = 6 (10.7%) had ongoing treatment at the time of analysis. The final number of 45 remaining patients was used to examine LTBI treatment outcomes (completion vs. non-completion).

Of those 45 patients, *n* = 42 (93.3%) completed their respective LTBI treatment. Reasons for non-completion included being lost to follow-up (*n* = 2 or 4.4%) and treatment discontinued before completion with reasons not documented in the chart (*n* = 1 or 2.2%).

Treatment completion outcome did not significantly vary by age, gender, categories of refugees, WHO region of origin, or year of arrival to Canada (*p* >0.05). Outcomes were also analyzed for different LTBI treatment regimens (3HP, 3HR, 4R, and 9H), and no statistically significant difference was found between these four groups (*p* > 0.05).

Table 3 outlines the outcomes by demographic data for treatment acceptance and completion and treatment completion by each LTBI treatment regimen with their associated respective value of ps.

As outlined in the previous section, LTBI screening test positivity was 23.9% (*n* = 125 positive tests out of *n* = 523 patients screened). Screening test positivity for IGRA and TSTs specifically were 22.3% (*n* = 117) and 24.2% (*n* = 8), respectively.

## 5. Discussion

This study sought to evaluate LTBI screening and treatment in our local refugee population, with care delivered by a specialized refugee clinic and additional community support. The Canadian TB standards indicate the minimal acceptable LTBI treatment delivery outcomes as follows: 80% of those eligible for LTBI prophylaxis accept treatment, and of those who accept treatment, at least 80% complete treatment (30). In our population, treatment acceptance was 84.8%, and treatment completion was greater than 90%, regardless of the regimen chosen. These rates compare favorably to other previous estimates in Canada. A study conducted in Edmonton in a refugee clinic implementing systematic LTBI screening of GARs found that 96% accepted treatment and 73% completed therapy with no difference noted between 9H and 4R regimens (31). Of the 45 eligible patients at BridgeCare Clinic in Winnipeg, 75% accepted therapy for LTBI and 79% completed 9H (32).

Recently, with shorter TB treatment regimens being preferred, LTBI treatment completion rates have generally improved. Another study done at BridgeCare examined their TB program outcomes with 4R and 3HP LTBI treatment regimens (33). Their reported treatment acceptance and completion rates were both 90%, a notable improvement compared to the previous study. LTBI treatment completion rates at REACH showed a trend of shorter treatment regimens having higher completion rates (despite no statistical difference between regimens with value of ps >0.05); though the numbers were small, treatment with 9H was still associated with a completion rate of >90%.

Interestingly, treatment acceptance was statistically different between adults and children (*p* = 0.02). Although underlying explanations for this difference were not explored, children are generally at higher risk of developing active TB (23) compared to adults, and this would have been discussed with caregivers, perhaps leading them to favor treatment initiation. Other Canadian studies mentioned above did not include pediatric-specific data.

Integrated patient-centered refugee care is another factor that has been acknowledged as being important in optimizing LTBI treatment success (34). At the REACH clinic, the medical team has adopted a multidisciplinary approach by working closely with nursing, in-house pharmacy, laboratory medicine, and medical imaging to create a medical home for the patients. The primary care team at REACH is responsible for TB screening and treatment, which allows patients to have sustained contact with their primary care providers and continuity of care as it relates to integrating LTBI management with ongoing medical care. The use of trained interpreters at every visit and medical staff espousing principles of culturally sensitive care are other aspects of patient-centered care at REACH. Finally, a vital asset has been the partnership with local settlement agencies and their case workers, who help coordinate care and remove barriers for patients, including language, transportation, medication funding, health literacy, and lack of familiarity with the healthcare system. The care workers from the settlement agencies act as community health workers (CHWs) and form an essential part of the clinic’s care team. This emphasis on relational care and supporting vulnerable people has been well studied in the treatment of chronic conditions in resource-poor areas (35) and could be a key strategy in reducing TB incidence in North America as well.

Historically, there has been some skepticism regarding the effectiveness of identifying and treating LTBI cases among refugees and immigrants resettling in low-incidence countries (36). However, this study and others (31–33) illustrate that systematic LTBI screening and treatment of refugees can be done in dedicated clinics with expertise and community support and that Canadian TB standards for acceptance and completion rates can be attained. Furthermore, this model has proven to be cost-beneficial in a refugee clinic in Montreal, with every dollar spent on LTBI treatment saving 2 dollars in averted active TB treatment (37). Most importantly, in the 12 years since opening their clinic, TB incidence among migrants screened at the clinic had decreased by 61%. In another study of a large community health center caring for over 124,000 patients, of which a large proportion comprised non-US-born immigrants, a number of electronic record and education interventions were implemented in order to reduce barriers to LTBI identification and management (38). These led to increased LTBI screening and treatment over a 10-year period. It offers yet another example of how primary care can help drive TB elimination in low-incidence countries such as Canada and the US.

Having said that, the challenges encountered at REACH over the last several years have been numerous, largely involving the need for more resources. Given the complexity of patient difficulties being discussed, communication through interpreters, and the need to get some sense of the patients’ understanding and experience of health issues, appointment times at REACH tend to be longer and often require multiple visits. All levels of staff at the clinic need to be mindful of the diverse health literacy skills of the patients, which requires continued effort and education. The pharmacy, in particular, has had to refine its communication and problem-solving skills as the potential for medication errors is significant. Given the importance of the CHWs at the settlement agencies and their role in facilitating patient care, communication between them and the clinic physicians must be direct and timely. This level of coordination and teamwork requires regular meetings to discuss successes and, more importantly, to troubleshoot challenges such as missed appointments, increasing in-person interpreters, and coordination of healthcare services.

Our study population’s LTBI test positivity was 23.9% overall. This is lower than previous reports from refugee clinics in Canada, which had prevalence rates ranging from 34 to 53% (31). However, during the study period, at least half of the patients that were seen at the clinic were from the eastern Mediterranean, a region described by the WHO as having a TB incidence of 112/100,000 (compared to 226/100,000 in sub-Saharan Africa) (34). A study of Syrian newcomers to Canada showed an LTBI prevalence rate of 9% (39). Significant variability in reported TB prevalence rates is to be expected based on the years of arrival and countries of origin (40).

Screening and treatment of LTBI among refugee patients can be an effective contribution to the goal of TB elimination in low-incidence countries. The REACH clinic espouses a multidisciplinary approach addressing barriers to care and a commitment to patient-centeredness and continuity of care. Given that TB is a disease rooted in social inequity, this emphasis on relational care and community support of vulnerable patients is critical. The shorter duration of LTBI treatment regimens is also contributing to high completion rates and, in combination with specialized refugee care, represents a path forward for LTBI management among this population.

## 6. Conceptual and methodological constraints

This study has a number of limitations. The retrospective design and chart review methodology bring their usual constraints, including missing or incomplete documentation and a relatively small number of participants, especially in the 3HP treatment group. Our study window was during a time when preferred treatment regimens were shifting, and therefore, we may not have captured differences in treatment completion outcomes among treatment regimens. The study period also occurred during the COVID-19 pandemic, thus reducing new refugee arrivals to Saskatoon. Because of our small sample size, the findings may not be reproducible or generalizable to other settings. Certainly, a qualitative study examining patients’ experience of care at REACH and, more specifically, concerning LTBI management would give us helpful insights into how treatment acceptance and completion could be improved further and offer avenues for future research. Furthermore, exploration of pediatric qualitative data in terms of treatment acceptance and why they contrast among adults would be valuable.

## Data availability statement

The raw data supporting the conclusions of this article will be made available by the authors, without undue reservation.

## Ethics statement

The studies involving humans were approved by the University of Saskatchewan Biomedical Ethics Board (Bio-REB ID number 2616). The studies were conducted in accordance with the local legislation and institutional requirements. Written informed consent for participation was not required from the participants or the participants’ legal guardians/next of kin in accordance with the national legislation and institutional requirements.

## Author contributions

All authors listed have made a substantial, direct, and intellectual contribution to the work and approved it for publication.
